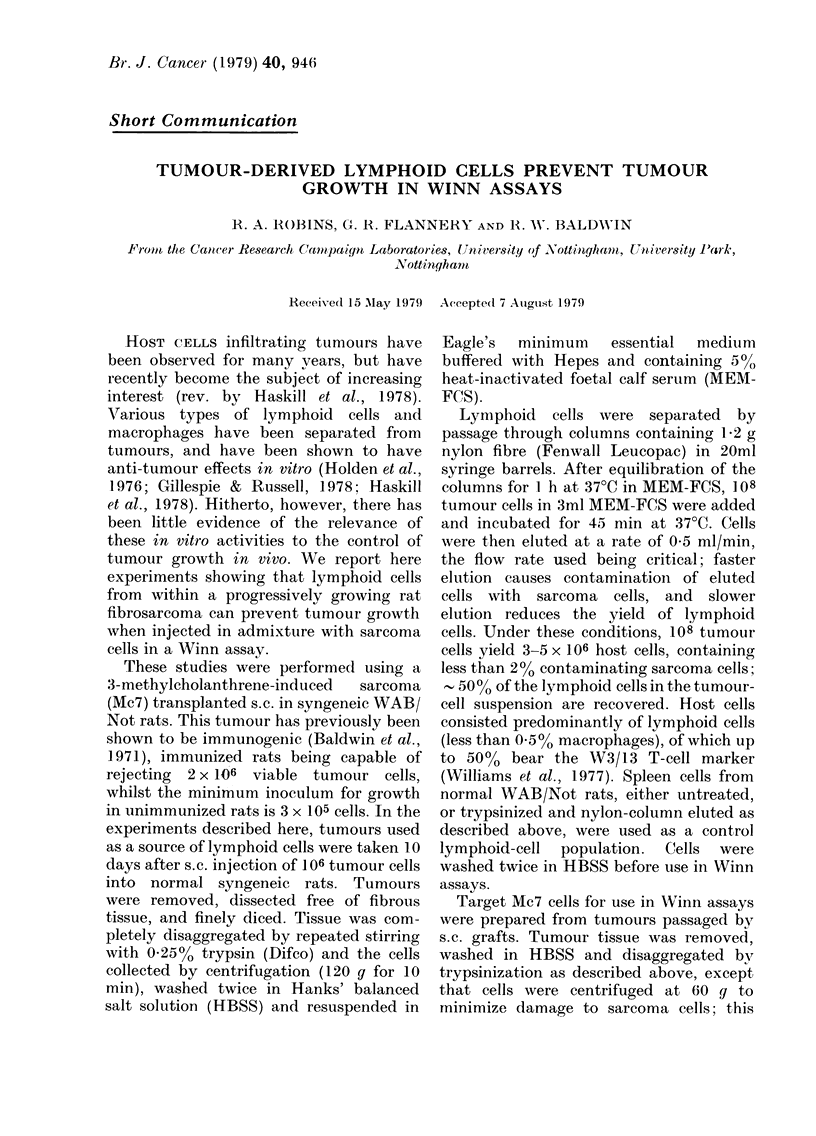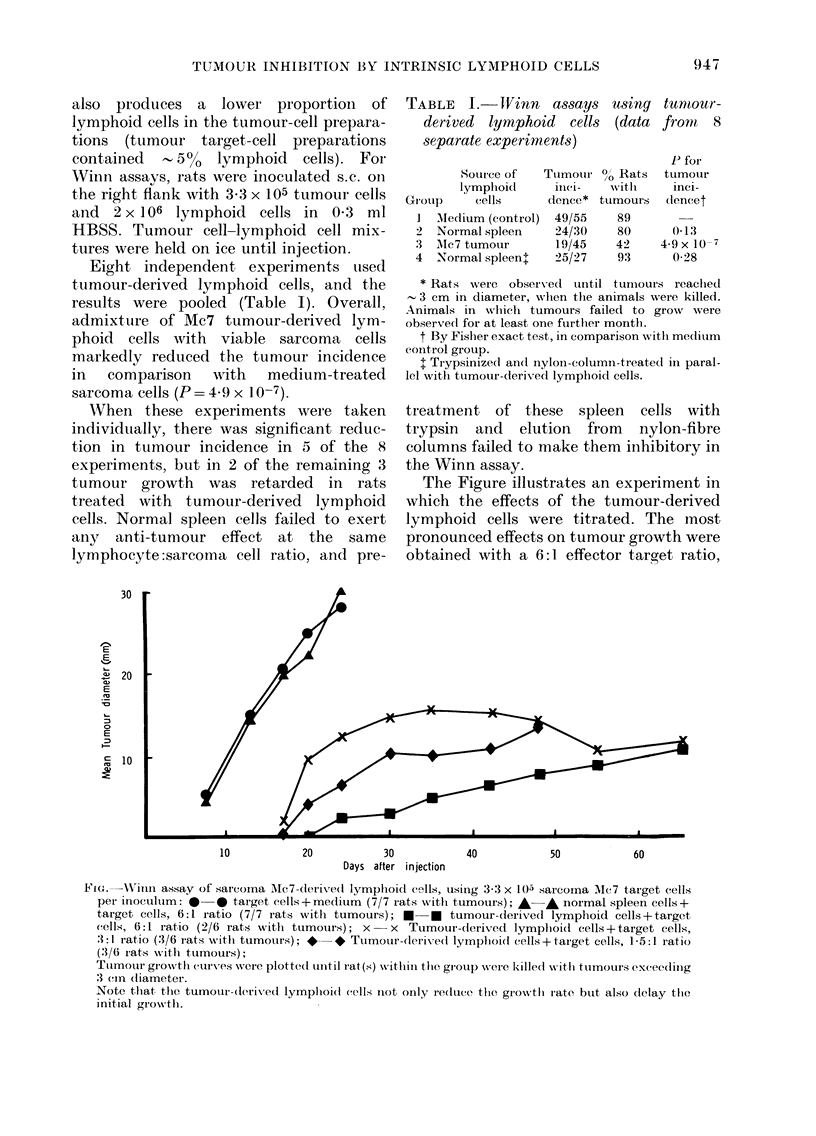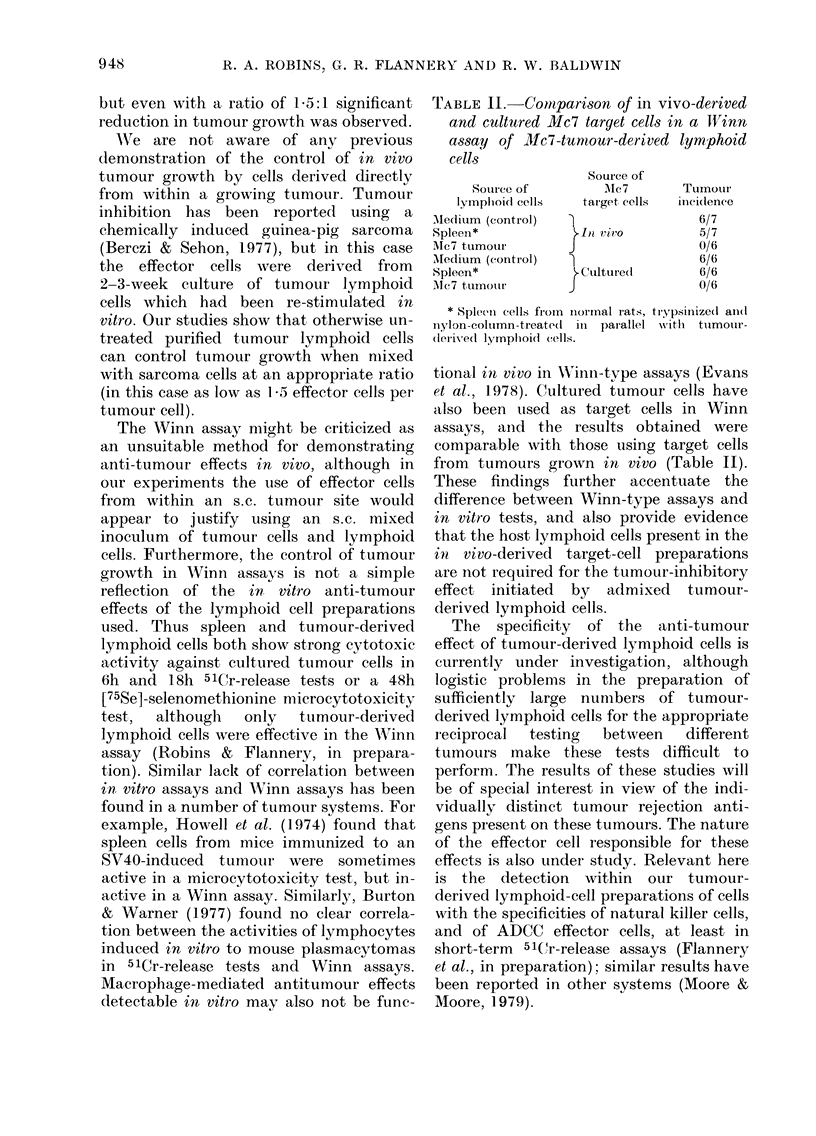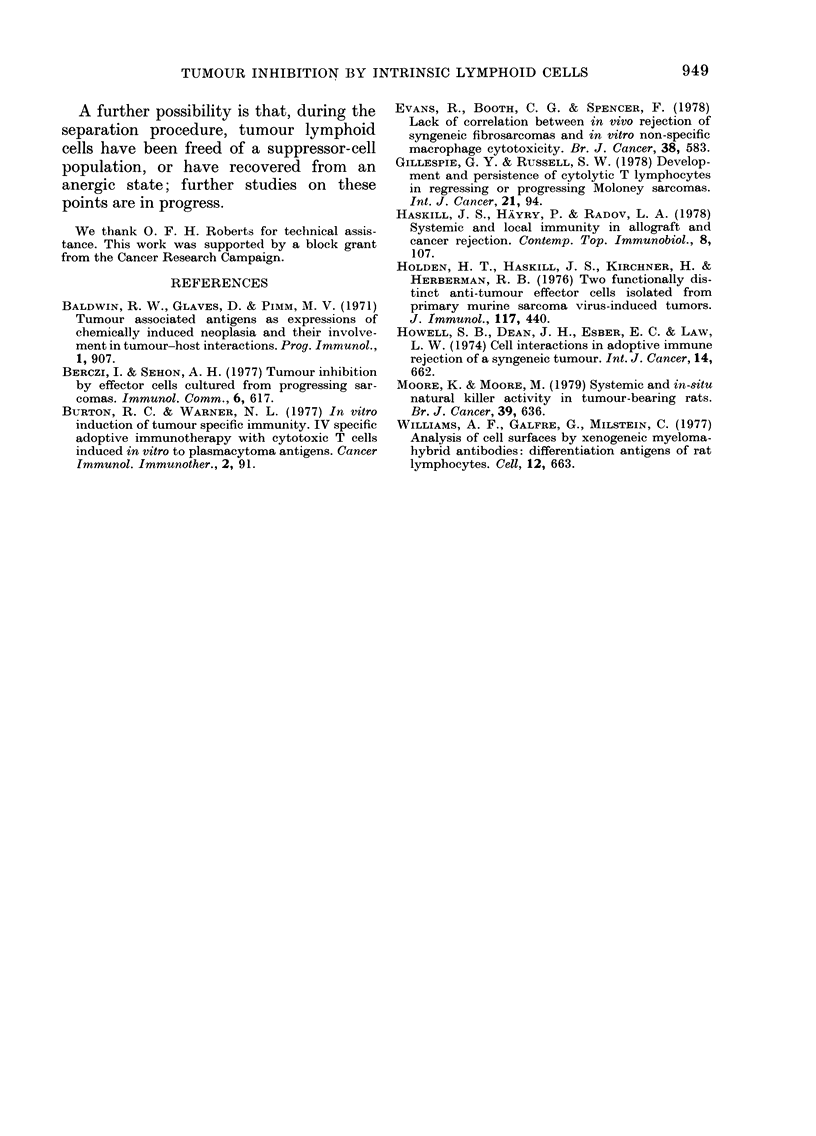# Tumour-derived lymphoid cells prevent tumour growth in Winn assays.

**DOI:** 10.1038/bjc.1979.290

**Published:** 1979-12

**Authors:** R. A. Robins, G. R. Flannery, R. W. Baldwin


*Br. J. Cancer* (1979) **40**, 946

***Short Communication***

### TUMOUR-DERIVED LYMPHOID CELLS PREVENT TUMOUR GROWTH IN WINN ASSAYS

R. A. ROBINS, G. R. FLANNERY AND R. W. BALDWIN

*From the Cancer Research Campaign Laboratories, University of Nottingham, University Park, Nottingham*

Received 15 May 1979 Accepted 7 August 1979

HOST CELLS infiltrating tumours have been observed for many years, but have recently become the subject of increasing interest (rev. by Haskill *et al.*, 1978). Various types of lymphoid cells and macrophages have been separated from tumours, and have been shown to have anti-tumour effects *in vitro* (Holden *et al.*, 1976; Gillespie & Russell, 1978; Haskill *et al.*, 1978). Hitherto, however, there has been little evidence of the relevance of these *in vitro* activities to the control of tumour growth *in vivo*. We report here experiments showing that lymphoid cells from within a progressively growing rat fibrosarcoma can prevent tumour growth when injected in admixture with sarcoma cells in a Winn assay.

These studies were performed using a 3-methylcholanthrene-induced sarcoma (Mc7) transplanted s.c. in syngeneic WAB/Not rats. This tumour has previously been shown to be immunogenic (Baldwin *et al.*, 1971), immunized rats being capable of rejecting $2 \times 10^6$ viable tumour cells, whilst the minimum inoculum for growth in unimmunized rats is $3 \times 10^5$ cells. In the experiments described here, tumours used as a source of lymphoid cells were taken 10 days after s.c. injection of $10^6$ tumour cells into normal syngeneic rats. Tumours were removed, dissected free of fibrous tissue, and finely diced. Tissue was completely disaggregated by repeated stirring with 0·25% trypsin (Difco) and the cells collected by centrifugation (120 *g* for 10 min), washed twice in Hanks' balanced salt solution (HBSS) and resuspended in Eagle's minimum essential medium buffered with Hepes and containing 5% heat-inactivated foetal calf serum (MEM-FCS).

Lymphoid cells were separated by passage through columns containing 1·2 g nylon fibre (Fenwall Leucopac) in 20ml syringe barrels. After equilibration of the columns for 1 h at 37°C in MEM-FCS, $10^8$ tumour cells in 3ml MEM-FCS were added and incubated for 45 min at 37°C. Cells were then eluted at a rate of 0·5 ml/min, the flow rate used being critical; faster elution causes contamination of eluted cells with sarcoma cells, and slower elution reduces the yield of lymphoid cells. Under these conditions, $10^8$ tumour cells yield $3–5 \times 10^6$ host cells, containing less than 2% contaminating sarcoma cells; $\sim 50\%$ of the lymphoid cells in the tumour-cell suspension are recovered. Host cells consisted predominantly of lymphoid cells (less than 0·5% macrophages), of which up to 50% bear the W3/13 T-cell marker (Williams *et al.*, 1977). Spleen cells from normal WAB/Not rats, either untreated, or trypsinized and nylon-column eluted as described above, were used as a control lymphoid-cell population. Cells were washed twice in HBSS before use in Winn assays.

Target Mc7 cells for use in Winn assays were prepared from tumours passaged by s.c. grafts. Tumour tissue was removed, washed in HBSS and disaggregated by trypsinization as described above, except that cells were centrifuged at 60 *g* to minimize damage to sarcoma cells; this also produces a lower proportion of lymphoid cells in the tumour-cell preparations (tumour target-cell preparations contained ~5% lymphoid cells). For Winn assays, rats were inoculated s.c. on the right flank with $3 \cdot 3 \times 10^5$ tumour cells and $2 \times 10^6$ lymphoid cells in 0·3 ml HBSS. Tumour cell–lymphoid cell mixtures were held on ice until injection.

Eight independent experiments used tumour-derived lymphoid cells, and the results were pooled (Table I). Overall, admixture of Mc7 tumour-derived lymphoid cells with viable sarcoma cells markedly reduced the tumour incidence in comparison with medium-treated sarcoma cells ($P = 4 \cdot 9 \times 10^{-7}$).

When these experiments were taken individually, there was significant reduction in tumour incidence in 5 of the 8 experiments, but in 2 of the remaining 3 tumour growth was retarded in rats treated with tumour-derived lymphoid cells. Normal spleen cells failed to exert any anti-tumour effect at the same lymphocyte:sarcoma cell ratio, and pre-

TABLE I.—*Winn assays using tumour-derived lymphoid cells (data from 8 separate experiments)*

| Group | Source of lymphoid cells | Tumour inci-dence* | % Rats with tumours | P for tumour inci-dence† |
|---|---|---|---|---|
| 1 | Medium (control) | 49/55 | 89 | — |
| 2 | Normal spleen | 24/30 | 80 | 0·13 |
| 3 | Mc7 tumour | 19/45 | 42 | $4 \cdot 9 \times 10^{-7}$ |
| 4 | Normal spleen‡ | 25/27 | 93 | 0·28 |

* Rats were observed until tumours reached ~3 cm in diameter, when the animals were killed. Animals in which tumours failed to grow were observed for at least one further month.

† By Fisher exact test, in comparison with medium control group.

‡ Trypsinized and nylon-column-treated in parallel with tumour-derived lymphoid cells.

treatment of these spleen cells with trypsin and elution from nylon-fibre columns failed to make them inhibitory in the Winn assay.

The Figure illustrates an experiment in which the effects of the tumour-derived lymphoid cells were titrated. The most pronounced effects on tumour growth were obtained with a 6:1 effector target ratio,

FIG.—Winn assay of sarcoma Mc7-derived lymphoid cells, using $3 \cdot 3 \times 10^5$ sarcoma Mc7 target cells per inoculum: ●—● target cells + medium (7/7 rats with tumours); ▲—▲ normal spleen cells + target cells, 6:1 ratio (7/7 rats with tumours); ■—■ tumour-derived lymphoid cells + target cells, 6:1 ratio (2/6 rats with tumours); ×—× Tumour-derived lymphoid cells + target cells, 3:1 ratio (3/6 rats with tumours); ◆—◆ Tumour-derived lymphoid cells + target cells, 1·5:1 ratio (3/6 rats with tumours);

Tumour growth curves were plotted until rat(s) within the group were killed with tumours exceeding 3 cm diameter.

Note that the tumour-derived lymphoid cells not only reduce the growth rate but also delay the initial growth.

but even with a ratio of 1·5:1 significant reduction in tumour growth was observed.

We are not aware of any previous demonstration of the control of *in vivo* tumour growth by cells derived directly from within a growing tumour. Tumour inhibition has been reported using a chemically induced guinea-pig sarcoma (Berczi & Sehon, 1977), but in this case the effector cells were derived from 2–3-week culture of tumour lymphoid cells which had been re-stimulated *in vitro*. Our studies show that otherwise untreated purified tumour lymphoid cells can control tumour growth when mixed with sarcoma cells at an appropriate ratio (in this case as low as 1·5 effector cells per tumour cell).

The Winn assay might be criticized as an unsuitable method for demonstrating anti-tumour effects *in vivo*, although in our experiments the use of effector cells from within an s.c. tumour site would appear to justify using an s.c. mixed inoculum of tumour cells and lymphoid cells. Furthermore, the control of tumour growth in Winn assays is not a simple reflection of the *in vitro* anti-tumour effects of the lymphoid cell preparations used. Thus spleen and tumour-derived lymphoid cells both show strong cytotoxic activity against cultured tumour cells in 6h and 18h $^{51}$Cr-release tests or a 48h [$^{75}$Se]-selenomethionine microcytotoxicity test, although only tumour-derived lymphoid cells were effective in the Winn assay (Robins & Flannery, in preparation). Similar lack of correlation between *in vitro* assays and Winn assays has been found in a number of tumour systems. For example, Howell *et al.* (1974) found that spleen cells from mice immunized to an SV40-induced tumour were sometimes active in a microcytotoxicity test, but inactive in a Winn assay. Similarly, Burton & Warner (1977) found no clear correlation between the activities of lymphocytes induced *in vitro* to mouse plasmacytomas in $^{51}$Cr-release tests and Winn assays. Macrophage-mediated antitumour effects detectable *in vitro* may also not be functional *in vivo* in Winn-type assays (Evans *et al.*, 1978). Cultured tumour cells have also been used as target cells in Winn assays, and the results obtained were comparable with those using target cells from tumours grown *in vivo* (Table II). These findings further accentuate the difference between Winn-type assays and *in vitro* tests, and also provide evidence that the host lymphoid cells present in the *in vivo*-derived target-cell preparations are not required for the tumour-inhibitory effect initiated by admixed tumour-derived lymphoid cells.

TABLE II.—*Comparison of* in vivo-*derived and cultured Mc7 target cells in a Winn assay of Mc7-tumour-derived lymphoid cells*

| Source of lymphoid cells | Source of Mc7 target cells | Tumour incidence |
|---|---|---|
| Medium (control) | *In vivo* | 6/7 |
| Spleen* | *In vivo* | 5/7 |
| Mc7 tumour | *In vivo* | 0/6 |
| Medium (control) | Cultured | 6/6 |
| Spleen* | Cultured | 6/6 |
| Mc7 tumour | Cultured | 0/6 |

* Spleen cells from normal rats, trypsinized and nylon-column-treated in parallel with tumour-derived lymphoid cells.

The specificity of the anti-tumour effect of tumour-derived lymphoid cells is currently under investigation, although logistic problems in the preparation of sufficiently large numbers of tumour-derived lymphoid cells for the appropriate reciprocal testing between different tumours make these tests difficult to perform. The results of these studies will be of special interest in view of the individually distinct tumour rejection antigens present on these tumours. The nature of the effector cell responsible for these effects is also under study. Relevant here is the detection within our tumour-derived lymphoid-cell preparations of cells with the specificities of natural killer cells, and of ADCC effector cells, at least in short-term $^{51}$Cr-release assays (Flannery *et al.*, in preparation); similar results have been reported in other systems (Moore & Moore, 1979).

A further possibility is that, during the separation procedure, tumour lymphoid cells have been freed of a suppressor-cell population, or have recovered from an anergic state; further studies on these points are in progress.

We thank O. F. H. Roberts for technical assistance. This work was supported by a block grant from the Cancer Research Campaign.

### REFERENCES

BALDWIN, R. W., GLAVES, D. & PIMM, M. V. (1971) Tumour associated antigens as expressions of chemically induced neoplasia and their involvement in tumour–host interactions. *Prog. Immunol.*, **1**, 907.

BERCZI, I. & SEHON, A. H. (1977) Tumour inhibition by effector cells cultured from progressing sarcomas. *Immunol. Comm.*, **6**, 617.

BURTON, R. C. & WARNER, N. L. (1977) *In vitro* induction of tumour specific immunity. IV specific adoptive immunotherapy with cytotoxic T cells induced *in vitro* to plasmacytoma antigens. *Cancer Immunol. Immunother.*, **2**, 91.

EVANS, R., BOOTH, C. G. & SPENCER, F. (1978) Lack of correlation between *in vivo* rejection of syngeneic fibrosarcomas and *in vitro* non-specific macrophage cytotoxicity. *Br. J. Cancer*, **38**, 583.

GILLESPIE, G. Y. & RUSSELL, S. W. (1978) Development and persistence of cytolytic T lymphocytes in regressing or progressing Moloney sarcomas. *Int. J. Cancer*, **21**, 94.

HASKILL, J. S., HÄYRY, P. & RADOV, L. A. (1978) Systemic and local immunity in allograft and cancer rejection. *Contemp. Top. Immunobiol.*, **8**, 107.

HOLDEN, H. T., HASKILL, J. S., KIRCHNER, H. & HERBERMAN, R. B. (1976) Two functionally distinct anti-tumour effector cells isolated from primary murine sarcoma virus-induced tumors. *J. Immunol.*, **117**, 440.

HOWELL, S. B., DEAN, J. H., ESBER, E. C. & LAW, L. W. (1974) Cell interactions in adoptive immune rejection of a syngeneic tumour. *Int. J. Cancer*, **14**, 662.

MOORE, K. & MOORE, M. (1979) Systemic and *in-situ* natural killer activity in tumour-bearing rats. *Br. J. Cancer*, **39**, 636.

WILLIAMS, A. F., GALFRE, G., MILSTEIN, C. (1977) Analysis of cell surfaces by xenogeneic myeloma-hybrid antibodies: differentiation antigens of rat lymphocytes. *Cell*, **12**, 663.